# ‘Taking Time’ – Extended mental health consultations in a deprived urban general practice: A retrospective descriptive study

**DOI:** 10.1371/journal.pone.0349885

**Published:** 2026-05-28

**Authors:** Siobhan Hinchy, Eimear Pilkington, Helen Tobin, Nandakumar Ravichandran, Walter Cullen, Brian O’Donoghue, Bobby Smyth, Anna Beug

**Affiliations:** 1 UCD Coombe Family Practice, University College Dublin, Dublin, Ireland; 2 School of Medicine, University College Dublin, Dublin, Ireland; 3 Trinity College Dublin, Dublin, Ireland; NYU Grossman School of Medicine: New York University School of Medicine, UNITED STATES OF AMERICA

## Abstract

**Background:**

Mental health presentations are common in general practice, particularly in socially deprived areas, yet standard consultation times often limit GPs’ ability to comprehensively assess and manage patients presenting with diverse emotional concerns. Such work is also difficult to capture in research, which often relies on coded diagnoses or prescribing data. This study aimed to describe the nature, management, and outcomes of extended mental health consultations in a deprived urban Irish practice.

**Methods:**

A retrospective descriptive secondary analysis was conducted on clinical charts for 195 patients who attended 30-minute extended mental health consultations between March 2021 and March 2023. Quantitative data on demographics, presenting complaints, prescriptions, and referrals were analysed descriptively, while clinical notes were systematically categorised to explore the spectrum of emotional concerns.

**Results:**

The median patient age was 29.0 years; 63.1% were female, and 69.8% lived in areas below the national average for social deprivation. Anxiety (47.7%) and low mood (42.1%) were the most common presenting complaints. Psychotropic prescriptions were initiated or modified for 37.9% of patients, with SSRIs the most prescribed. Of all patients, 26.7% were referred to psychiatric services and 57.4% were signposted to community mental health supports.

**Conclusion:**

The study demonstrated GPs in this deprived urban practice encountered wide range of mental health concerns across all ages and managed substantial proportion entirely within the community. These findings highlight the potential for delivering mental health care in general practice when time and resources are sufficient and underscore the need for better access to comprehensive primary care mental health supports.

## Introduction

Mental health disorders (MHD) represent a significant proportion of presentations in general practice [1]. In Ireland, more than 25% of the consultations in general practice pertain to a mental health issue and the majority of these are managed exclusively at the primary care level [2,3]. A study among general practitioners (GPs) reported that fewer than 5% of patients presenting with mental health difficulties were referred to a specialist [4]. These presentations are often heterogeneous, undifferentiated and result from a complex mixture of biopsychosocial factors [5,6].

Although general practice plays an important role in managing mental health conditions, significant barriers to the provision of mental health care in the community exist. These barriers include a lack of designated funding and a lack of time to discuss mental health problems during consultations [7–11]. GPs working in deprived populations face additional challenges due to workload challenges and capitation payments [12].

Research shows that the prevalence of psychological distress increases with the level of social deprivation. At the same time, average consultation times in practices in more deprived areas tend to be shorter due to high demand, meaning that patients who may need longer consultations and enhanced care are less likely to receive them [13]. A 2023 study underscored the feasibility and value of extended consultations for patients with severe mental illness in general practice and highlighted the need for a longer follow-up for better implementation of such consultations [14].

A Landmark commission on Youth Mental health flagged a crisis in youth mental health with a surge in demand for youth mental health care over the last 10 years, with little to no increase in service provision [15]. In Ireland, The Mental Health Commission document published in 2023 flagged concerns regarding the capacity of tertiary services to provide a safe and therapeutic service for young people with mental health difficulties [16].

In response to a perceived increased incidence of patients across the life span seeking mental health support in general practice, an extended mental health consultation type was piloted in a single general practice in a deprived urban area of Dublin, Ireland. This initiative supported by the Health Service Executive aimed to provide extra time to clinicians to engage with complex mental health presentations [17].

This consultation model considered the patient–GP relationship to be therapeutic and valued continuity of care. It incorporated a shared understanding of presentations, facilitated by open questioning from the GP, which helped patients reflect on and clarify their experiences [18].

This paper presents the findings of a two-year, single-practice study of extended mental health consultations in a deprived urban area of Dublin, Ireland.

The aims of the extended mental health consultations included

- To describe the range and complexity of mental health presentations in deprived primary care, when cases were selected not based on a coded diagnosis, prescription or referral, but on the judgement of a GP that extra time was needed to assess and treat the patient.
- To examine the content of these extended mental health consultations in terms of the presenting complaints, patient demographics and outcomes such as referral, prescribing and use of secondary care.
- To examine the proportion of the extended mental health consultations given to different age groups, considering current concerns about the burden of youth mental health presentations to primary care.

## Methods

### Procedure

We conducted a retrospective descriptive study using secondary analysis of clinical charts. Quantitative data, including demographics, presenting complaints, prescribing, and referrals, were extracted and analysed descriptively. To capture the range of presentations and outcomes observed in practice, clinical information from free-text notes was systematically categorised into clinically relevant groups. This approach was informed by established frameworks for observational health data analysis and WHO Data Quality Review guidelines, which guided data extraction, cleaning, and validation [19].

### Interventional design and delivery

Extended 30-minute consultations were offered at the GP’s discretion to patients presenting with emotional difficulties or mental health concerns likely to benefit from additional time. A significant number of these patients presented initially in regular consultations where the mental health concern was only one of several issues. In such cases, GPs were encouraged to arrange an extended mental health consultation as a review appointment, rather than immediately, so the difficulties could be explored in more detail. The primary aim was to offer these appointments to patients presenting with more severe mental health difficulties. No formal screening tool, diagnostic threshold, or referral criteria were applied in the selection of patients for extended consultations. Allocation was based solely on GP clinical judgement, reflecting the study’s intention to capture the full spectrum of mental health presentations encountered in primary care, beyond those identifiable through diagnostic coding or prescribing data alone. In practice, decisions were based on clinical assessment of complexity, risk (including self-harm or suicidality), and the presence of significant psychosocial distress requiring additional consultation time.

### Setting, recruitment and delivery

The consultations were piloted in a single general practice in a deprived urban area of Dublin serving a population of approximately 2400 General Medical Services (GMS) patients and 2600 private patients, where patients frequently reported challenges accessing primary care and community mental health services. In the Irish health system, access to general practitioners (GPs) is mixed between public and private models. Eligibility for the General Medical Services (GMS) scheme is means-tested and entitles patients to free GP care, prescriptions (subject to a small co-payment), and other primary care services. A less comprehensive option, the Doctor’s Visit Card (DVC), provides free GP consultations but not prescription coverage. Patients without GMS or DVC eligibility are required to pay out of pocket for GP visits, with consultation fees typically ranging from €50 to €70 per visit. This mixed system can create financial and structural barriers to timely access, particularly for patients in socioeconomically deprived areas.

No formal training protocol was provided to participating clinicians. GPs were advised at a practice level to use extended appointments judiciously, reserving them for more severe or complex presentations where additional consultation time was felt to be of clinical benefit. Allocation remained at GP discretion, reflecting the study’s intention to capture the breadth of complex mental health presentations encountered in primary care. GPs were not restricted from offering more than one extended consultation to each patient. Although GPs could provide multiple extended consultations per patient, the data reported in this paper are patient-based rather than consultation-based.

### Ethical approvals

Ethics approval was obtained from the Irish College of General Practitioners Research Ethics Committee (Ref: ICGP_REC_2023_007). The study was conducted as a retrospective service evaluation using existing clinical records, with no additional assessments or patient contact. As all data were fully pseudonymised prior to analysis and presented in aggregated format, the requirement for informed consent was waived by the Ethics Committee; therefore, no written or verbal consent was sought from patients. All data presented were anonymised and aggregated across age groups. The retrospective anonymised dataset was accessed on 12 October 2023 for research purposes. No identifiable information was available to the authors at any stage during or after data collection.

### Data collection

A report was generated of all extended mental health consultations during the study period. Researchers (SH, EP, HT) extracted demographic and prescribing data from the individual patient charts of all 195 identified patients. Clinical information relating to presentations, referrals, and outcomes was extracted from free-text notes and grouped into clinically meaningful categories using an iterative, team-based approach. To ensure consistency in categorisation, data extraction was performed by SH, EP, and HT, with any discrepancies resolved through discussion with AB. Following data extraction, all patient identifiers were removed, and the dataset was pseudonymised and aggregated prior to analysis. Quantitative analysis was performed by NR, HT, and SH. Patients were classified into different areas of deprivation using Ireland’s Pobal HP Deprivation Index, which is calculated at the Small Area level geographic units of approximately 80–120 households nested within electoral divisions [20].

### Data analysis

All data were presented in aggregated form across five age groups: Child (aged 4–12), Adolescent (aged 12–17), Young adult (aged 18–25), Adult (aged 25–70) and older adult (aged over 70). Descriptive statistical analysis was performed using SPSS. Categorised clinical data were summarised using frequencies and proportions across age groups.

## Results

### Description of participants

A total of 260 extended mental health consultations were conducted across 195 patients (mean 1.33 consultations per patient, range: 1–2) which were delivered by the general practitioners working in the practice between March 2021 and March 2023. Around 16 doctors participated in delivering extended consultations over the study period, including GP principals, fellows, academic staff and registrars, reflecting the multi-doctor composition of this urban training practice.

The median age of the 195 patients who attended the extended consultations was 29.0 years, ranging from 4 years to 86 years. Of the cohort, 63.1% (n = 123) were female and 36.4% (n = 71) were male. Of them, 12.3% (n = 24) were children aged 4–12 years, 10.8% (n = 21) were adolescents aged 12–17 years, 14.9% (n = 29) were young adults aged 18–25 years old, 55.4% (n = 108) were adults aged 25–70 years, 6.7% (n = 13) were older adults aged over 70 years. Of them, 66.3% (n = 129 patients) were registered as either GMS or DVC patients and 33.9% (n = 66 patients) were private patients. A total of 131 patients (69.8%) of the study participants lived in areas below the national average of social deprivation scores ranging from −20 to −9, indicating high degrees of disadvantage and 38 patients (20.3%) lived in marginally above average (score −9–0). The prescribing patterns, medications and referrals are presented in aggregated format by age cohorts.

Within each age group, 11 children (45.9%), 13 adolescents (62.0%), 20 young adults (69.0%), 71 adults (65.8%), and 8 older adults (61.6%) were female.

Within each age group, 17 children (70.9%), 73 adults (67.6%), and 12 older adults (92.4%) held GMS medical cards. In contrast, adolescents (62.0%) and young adults (58.7%) were more commonly private patients.

### Reasons for extended consultations

The most common presenting complaints overall were anxiety (93 patients, 47.7%), low mood (82 patients, 42.1%) and self-harm/suicidality (36 patients, 18.5%). However, these varied considerably by age cohort. These categories were derived from clinical documentation and reflect how presentations were recorded in routine practice.

In the child age cohort (aged 4–12), the most common presenting complaints were concerns about neurodivergence (41.7%), challenging behaviour (41.7%) and anxiety (37.5%).

Anxiety and low mood were common in all age cohorts. Among adults aged 25–70, anxiety and low mood were the most frequent presenting complaints, while psychosocial stressors including grief, work-related stress, interpersonal difficulties, social isolation, and housing concerns were also common (39 patients, 36.1%), as was substance misuse (20 patients, 18.6%). In contrast, psychosocial stressors represented the most frequent presentation among older adults (n = 8, 61.5%) (Table 1).

**Table 1:** Presenting complaint by age cohort (N = 195).

	Child (aged 4–12) (n = 24)	Adolescent (aged 12–17) (n = 21)	Young adult (aged 18–25) (n = 29)	Adult (aged 25–70) (n = 108)	Older adult (aged > 70) (n = 13)	N = 195
**Presenting complaint**						
Anxiety	9 (37.5%)	10 (47.7%)	18 (62.1%)	54 (50.0%)	2 (15.4%)	**93 (47.7%)**
Low mood	3 (12.5%)	12 (57.2%)	15 (51.8%)	49 (45.4%)	3 (23.1%)	**82 (42.1%)**
Self-harm/ suicide	4 (16.7%)	10 (47.7%)	4 (13.8%)	18 (16.7%)	0 (0.0%)	**36 (18.5%)**
Psychosocial stressors	0 (0.0%)	0 (0.0%)	0 (0.0%)	39 (36.1%)	8 (61.5%)	**47 (24.1%)**
Substance Misuse	0 (0.0%)	0 (0.0%)	0 (0.0%)	20 (18.6%)	1 (7.7%)	**21 (10.8%)**
Neurodivergence	10 (41.7%)	0 (0.0%)	1 (3.5%)	4 (3.8%)	0 (0.0%)	**15 (7.7%)**
Somatic symptoms	2 (8.4%)	1 (4.8%)	1 (3.5%)	8 (7.5%)	1 (7.7%)	**13 (6.7%)**
Challenging behaviour	10 (41.7%)	2 (9.6%)	1 (3.5%)	0 (0.0%)	0 (0.0%)	**13 (6.7%)**
Sleep problems	2 (8.4%)	0 (0.0%)	3 (10.4%)	4 (3.8%)	3 (23.1%)	**12 (6.2%)**
Obsessive compulsions	0 (0.0%)	1 (4.8%)	2 (6.9%)	8 (7.5%)	0 (0.0%)	**11 (5.6%)**
Psychotic symptoms	0 (0.0%)	0 (0.0%)	2 (6.9%)	4 (3.8%)	1 (7.7%)	**7 (3.6%)**
Eating disordered	1 (4.2%)	0 (0.0%)	4 (13.8%)	1 (1.0%)	0 (0.0%)	**6 (3.1%)**
School refusal	3 (12.5%)	2 (9.6%)	0 (0.0%)	0 (0.0%)	0 (0.0%)	**5 (2.6%)**
Memory concerns	0 (0.0%)	0 (0.0%)	0 (0.0%)	1 (1.0%)	3 (23.1%)	**4 (2.1%)**
Trauma	0 (0.0%)	0 (0.0%)	0 (0.0%)	4 (3.8%)	0 (0.0%)	**4 (2.1%)**
Gender identity	0 (0.0%)	1 (4.8%)	0 (0.0%)	2 (1.9%)	0 (0.0%)	**3 (1.5%)**
Mania/ hypomania	0 (0.0%)	0 (0.0%)	0 (0.0%)	2 (1.9%)	0 (0.0%)	**2 (1.0%)**
Paranoia	0 (0.0%)	0 (0.0%)	0 (0.0%)	1 (1.0%)	0 (0.0%)	**1 (0.5%)**

Note – patients could have multiple presenting complaints.

Of the 195 patients, 130 patients (66.7%) were not on psychotropic prescription at the time of their extended mental health consultation. Among older adults, six patients (46.2%) had an existing psychotropic prescription, compared to 51 adults (47.2%) and four young adults (13.8%).

Of the 195 patients, prescriptions were initiated or modified for 74 patients (37.9%). Within each age group, prescriptions were initiated for 18 young adults (62.1%), 50 adults (46.3%), and four older adults (30.8%). Of the 74 prescriptions, the most common change was starting new prescriptions (44 patients, 59.5%), while some other changes such as dose increases, switching medications and restarting prior medications were also found (see Table 2).

**Table 2:** Prescribing patterns by age cohort (N = 195).

	Child (aged 4–12) (n = 24)	Adolescent (aged 12–17) (n = 21)	Young adult (aged 18–25) (n = 29)	Adult (aged 25–70) (n = 108)	Older adult (aged > 70) (n = 13)	N = 195
**Existing prescriptions at the time of consultation**						
Previous psychotropic prescription	1 (4.2%)	3 (14.3%)	4 (13.8%)	51 (47.2%)	6 (46.2%)	**65 (33.3%)**
No previous psychotropic prescription	23 (95.8%)	18 (85.7%)	25 (86.2%)	57 (52.8%)	7 (53.8%)	**130 (66.7%)**
**Prescription initiation/ modification**						
	**Child** **(aged 4–12)** **(n = 24)**	**Adolescent** **(aged 12–17)** **(n = 21)**	**Young adult (aged 18–25)** **(n = 29)**	**Adult** **(aged 25–70)** **(n = 108)**	**Older adult (aged > 70)** **(n = 13)**	**N = 195**
Prescription initiated/ modified	0 (0.0%)	2 (9.5%)	18 (62.1%)	50 (46.3%)	4 (30.8%)	**74 (37.9%)**
Prescription not initiated/ modified	24 (100.0%)	19 (90.5%)	11 (37.9%)	58 (53.7%)	9 (69.2%)	**121 (62.1%)**
**Changes to prescriptions (N = 74)**						
	**Child** **(aged 4–12)** **(n = 0)**	**Adolescent** **(aged 12–17)** **(n = 2)**	**Young adult (aged 18–25)** **(n = 18)**	**Adult** **(aged 25–70)** **(n = 50)**	**Older adult (aged > 70)** **(n = 4)**	**N = 74**
Started	0 (0.0%)	2 (100.0%)	18 (100.0%)	23 (46.0%)	1 (25.0%)	**44 (59.5%)**
Restarted	0 (0.0%)	0 (0.0%)	0 (0.0%)	5 (10.0%)	0 (0.0%)	**5 (6.8%)**
Switched	0 (0.0%)	0 (0.0%)	0 (0.0%)	9 (18.0%)	0 (0.0%)	**9 (12.2%)**
Dose increased	0 (0.0%)	0 (0.0%)	0 (0.0%)	10 (20.0%)	2 (50.0%)	**12 (16.2%)**
Short term medication	0 (0.0%)	0 (0.0%)	0 (0.0%)	3 (6.0%)	1 (25.0%)	**4 (5.4%)**

Of the 195 patients, prescriptions were initiated or modified for 74 patients (37.9%). No children were prescribed any psychotropic medications, and two adolescents received prescriptions, both for sleep medication. Among the young adults who had their medications modified, 13 (44.8%) were prescribed Selective Serotonin Reuptake Inhibitors (SSRIs). There was more varied prescribing among general adults aged 25–70 years. Common prescriptions included 23 adults (21.3%) for SSRIs, 11 (10.2%) for mirtazapine and 6 (5.6%) for benzodiazepine. No patient prescribed more than one medication (Table 3).

**Table 3:** Prescriptions by age cohort (N = 195).

	Child (aged 4–12) (n = 24)	Adolescent (aged 12–17) (n = 21)	Young adult (aged 18–25) (n = 29)	Adult (aged 25–70) (n = 108)	Older adult (aged > 70) (n = 13)	N = 195
**Prescriptions**						
No prescriptions	24 (100.0%)	19 (90.5%)	11 (37.9%)	58 (53.7%)	9 (69.2%)	**121 (62.1%)**
SSRI	0 (0.0%)	0 (0.0%)	13 (44.8%)	23 (21.3%)	2 (15.4%)	**38 (19.5%)**
SNRI	0 (0.0%)	0 (0.0%)	0 (0.0%)	3 (2.8%)	0 (0.0%)	**3 (1.5%)**
Alcohol Detox	0 (0.0%)	0 (0.0%)	0 (0.0%)	1 (0.9%)	0 (0.0%)	**1 (0.5%)**
Amitryptiline	0 (0.0%)	0 (0.0%)	0 (0.0%)	1 (0.9%)	0 (0.0%)	**1 (0.5%)**
Benzodiazepine	0 (0.0%)	0 (0.0%)	2 (6.9%)	6 (5.6%)	1 (7.7%)	**9 (4.6%)**
Mirtazapine	0 (0.0%)	0 (0.0%)	0 (0.0%)	11 (10.2%)	0 (0.0%)	**11 (5.6%)**
Other sleeper	0 (0.0%)	2 (9.5%)	1 (3.4%)	0 (0.0%)	1 (7.7%)	**4 (2.1%)**
Antipsychotic	0 (0.0%)	0 (0.0%)	1 (3.4%)	3 (2.8%)	0 (0.0%)	**4 (2.1%)**
Quetiapine	0 (0.0%)	0 (0.0%)	0 (0.0%)	1 (0.9%)	0 (0.0%)	**1 (0.5%)**
Stimulant	0 (0.0%)	0 (0.0%)	0 (0.0%)	0 (0.0%)	0 (0.0%)	**0 (0.0%)**
Z Drug	0 (0.0%)	0 (0.0%)	1 (3.4%)	1 (0.9%)	0 (0.0%)	**2 (1.0%)**

Of the 195 patients, 52 patients (26.7%) were previously referred to Child and Adolescent Mental Health Services (CAMHS) or Community Mental Health Teams (CMHT).

Of the 195 patients, 143 (73.3%) were managed solely within primary care, while 52 (26.7%) received new psychiatry referrals. Of them, six were urgent referrals and one was involuntary admission. Notably, of the 52 patients referred, 35 patients (67.3%) were accepted by psychiatric services (Table 4).

**Table 4:** Psychiatry referral by age cohort (N = 195).

	Child (aged 4–12) (n = 24)	Adolescent (aged 12–17) (n = 21)	Young adult (aged 18–25) (n = 29)	Adult (aged 25–70) (n = 108)	Older adult (aged > 70) (n = 13)	N = 195
Previous Psychiatry CAMHS/ CMHT	3 (12.5%)	9 (42.9%)	15 (51.8%)	20 (18.6%)	5 (38.5%)	52 (26.7%)
**Psychiatry referrals (N = 195)**						
No new referrals	17 (70.9%)	12 (57.2%)	24 (82.8%)	78 (72.3%)	12 (92.4%)	143 (73.3%)
New Psych referral	7 (29.2%)	9 (42.9%)	5 (17.3%)	30 (27.8%)	1 (7.7%)	52 (26.7%)
**Types of referrals (N = 52)**						
Urgent referral	0 (0.0%)	0 (0.0%)	2 (6.9%)	4 (3.8%)	0 (0.0%)	6 (3.1%)
Involuntary admission	0 (0.0%)	0 (0.0%)	0 (0.0%)	1 (1.0%)	0 (0.0%)	1 (0.6%)
**Referral acceptance (N = 52)**						
Referral accepted	4 (57.1%)	7 (77.7%)	3 (60.0%)	20 (66.7%)	1 (100.0%)	**35 (67.3%)**

Of the 195 patients, 83 (42.6%) were not referred to any community services. Among the 112 patients (57.4%) who received referrals or signposting, the most common specialist mental health referral for adults was community counselling services. In young adults the most common services recommended were Jigsaw and Pieta house. In adults the most common services used were local low cost counselling services or CIPC (Counselling in primary care) or social prescribing [21]. Patients with primary addiction issues were signposted to HSE addiction services. Most services were self-referral and no official letter was required. General well-being advice (e.g., exercise, sleep hygiene) was not included. Multiple referrals were possible. For children, disability services (3, 12.5%) and medical referrals (2, 8.4%) were most common while addiction services were the second most common referral for adults (12 patients, 11.2%) (Table 5).

**Table 5:** Community referral or signposting by age cohort (N = 195).

	Child (aged 4–12) (n = 24)	Adolescent (aged 12–17) (n = 21)	Young adult (aged 18–25) (n = 29)	Adult (aged 25–70) (n = 108)	Older adult (aged > 70) (n = 13)	N = 195
**Referral type**						
No community referral or signposting	17 (70.9%)	10 (47.7%)	15 (51.8%)	33 (30.6%)	8 (61.6%)	**83 (42.6%)**
Family supports	1 (4.2%)	1 (4.8%)	0 (0.0%)	2 (1.9%)	0 (0.0%)	**4 (2.1%)**
Peer support group	0 (0.0%)	0 (0.0%)	1 (3.5%)	1 (1.0%)	0 (0.0%)	**2 (1.1%)**
Disability services	3 (12.5%)	0 (0.0%)	0 (0.0%)	0 (0.0%)	0 (0.0%)	**3 (1.6%)**
Online therapy	0 (0.0%)	0 (0.0%)	0 (0.0%)	6 (5.6%)	1 (7.7%)	**7 (3.6%)**
Social prescribing	0 (0.0%)	1 (4.8%)	0 (0.0%)	4 (3.8%)	0 (0.0%)	**5 (2.6%)**
HSE counselling services	1 (4.2%)	0 (0.0%)	1 (3.5%)	8 (7.5%)	0 (0.0%)	**10 (5.2%)**
Addiction services	0 (0.0%)	0 (0.0%)	0 (0.0%)	12 (11.2%)	0 (0.0%)	**12 (6.2%)**
Medical referral	2 (8.4%)	1 (4.8%)	1 (3.5%)	1 (1.0%)	2 (15.4%)	**7 (3.6%)**
Community counselling and services	1 (4.2%)	9 (42.9%)	11 (38.0%)	48 (44.5%)	2 (15.4%)	**71 (36.5%)**

## Discussion

### Key findings

This study provides novel insights into the nature and management of mental health consultations in Irish general practice. We found that most mental health presentations were managed entirely within primary care and community services. In keeping with the demographic of the practice, the majority of patients were from areas of social deprivation. Presentations varied significantly by age. Anxiety, low mood, and psychosocial stressors dominated the clinical picture, particularly among adults aged 25–70 years. Psychotropic medications were initiated or modified in 74 patients (37.9%) during the extended consultations studied, with selective serotonin reuptake inhibitors (SSRIs) comprising the majority of prescriptions (38 patients, 19.5%). An audit conducted on all patients attending this practice during the same study period reported that 40 young adults (7.6%) were on SSRIs across the practice. The relatively high prescribing rates found among young adults is notable and may reflect the tendency of this cohort to present late and during periods of acute distress, consistent with broader evidence of a youth mental health crisis [15,22,23]. Despite the range and complexity of these presentations, 143 patients (73.3%) were solely managed in primary care and only 52 patients (26.7%) generated a psychiatry referral. Of these referrals 67.3% were accepted.

### Comparison to existing literature

Our findings align with international evidence highlighting the central role of GPs in managing complex presentations and emotional distress in primary care [24]. The relatively high rates of prescribing amongst young adults may reflect a cohort who are more likely to seek mental health care when in crisis or be reflective of a “Youth mental health crisis”, in which young people experience heightened levels of psychological distress while simultaneously “ageing out” of child and adolescent services [15]. In the Irish context, referral criteria for secondary care may further drive pharmacological management: adults require documented trials of two antidepressants at optimal doses before referral, whereas this stipulation does not apply to those under 18 [4]. This distinction may inadvertently incentivise early prescribing in young adults in the context of limited access to broader multidisciplinary supports.

This study is, to our knowledge, the first in an Irish context to directly analyse extended mental health consultations where cases are identified on the basis of a special consultation type rather than coded diagnoses or prescribing data. Previous Irish research has demonstrated that even patients with severe and enduring mental illness are under-coded in general practice records [25]. GPs often report reluctance to code mental health problems due to diagnostic uncertainty and concern that coding may stigmatise patients [26].

Of the 195 patients, 143 (73.3%) were managed solely within primary care, while 52 (26.7%) received new psychiatry referrals. It is important to note that the proportion of patients referred to CAMHS/CMHT should not be regarded as a simple marker of success [14]. In some cases, the extended consultation may provide sufficient support and management within primary care, thereby avoiding unnecessary referral. In other cases, the extended consultation may uncover previously unrecognised concerns or risks, appropriately triggering referral to specialist services. Thus, both outcomes management within primary care and appropriate onward referral can be viewed as indicators of effective consultation [27].

Our observation that SSRIs are the most frequently prescribed psychotropic agents mirrors international trends, with increasing antidepressant prescribing noted across Europe and North America [28]. At the same time, benzodiazepine prescribing has declined significantly, consistent with restrictive guidance on their short-term use [29,30]. Our findings, therefore, capture a broader spectrum of emotional distress, from subclinical stressors to more severe presentations, which traditional data sources may overlook.

### Strengths and limitations

A key strength of this work is the prospective capture of extended consultations over two years, allowing a more nuanced picture of workload, prescribing, and referral practices in general practice. By including uncoded consultations, we avoided underestimating the actual workload of community mental health care. This single-centre observational design, focusing on self-selected patients presenting with emotional distress, limits the generalisability of findings. The lack of a control or comparison group restricts causal inference, and the findings may not reflect practices in other regions. No published studies were identified that report practice-level, within-same-period comparator outcomes for standard-length mental health consultations in Irish general practice that would allow direct comparison with the extended consultations in this study. But within the same practice population during the study period, an audit found that 7.6% of young adults were prescribed SSRIs, compared to higher prescribing rates observed in the extended consultation cohort.

As consultations were offered at the GP’s discretion, GPs may have preferentially selected patients perceived as more engaged, more complex, or more likely to benefit from extended time, which may influence the observed patterns of presentation and management. Furthermore, the reliance on routinely collected clinical data carries the possibility of missing information or misclassification. The broad adult age category (25–70 years) used in this analysis encompasses multiple life stages and may obscure clinically meaningful differences between subgroups within this range. This categorisation was retained due to sample size constraints but may limit detection of differences across life stages.

### Implications for future research, clinical practice and policy

These findings highlight the central role of general practice in addressing the mental health needs of socially deprived populations. The study underscores the value of extended consultations in capturing the breadth of emotional concerns that may often remain invisible in routine data. Future research should examine whether extended consultations improve patient and GP satisfaction, clinical outcomes, and healthcare utilisation compared with standard consultation lengths. Qualitative research exploring the lived experiences of patients and practitioners could also explore the barriers and enablers to delivering high-quality mental health care in general practice. Better recognition and resourcing of the mental health workload in general practice is essential. Scaling up extended consultation models across multiple practices and regions could provide a more representative understanding of patient needs and assess the feasibility of this approach in addressing gaps between rising mental health demand and limited specialist services. As there was no control group we could not directly comment on the relative value of extended mental health consultations, future research of this nature would be very valuable given the lack of any special resourcing of mental health care in Irish general practice.

## Conclusion

This study highlights that creating a dedicated mental health consultation type within a GP system allowed for a unique insight into the nature of mental health work in Irish primary care. Furthermore, implementing extended consultations in this deprived Irish general practice allowed for examination of the more complex work encountered. This research was able to capture the heterogeneous nature of mental health presentations, and important data on primary care management including prescribing, signposting and referral, some of which might be missed by more traditional research methods, and this study demonstrated the significant proportion of mental health presentations which can be solely managed within primary care. While this work does not allow us to comment directly on the relative value of extended consultations for mental health presentations it does point to the importance of more research in this area at a time where the demand for mental health care is increasing and pressures on secondary care services are creating significant limits to access. The impact of funded, longer consultation times for complex mental health care in general practice should be explored in future research.
